# Identifying stage-associated hub genes in bladder cancer via weighted gene co-expression network and robust rank aggregation analyses

**DOI:** 10.1097/MD.0000000000032318

**Published:** 2022-12-23

**Authors:** Fu Feng, Yu-Xiang Zhong, Jian-Hua Huang, Fu-Xiang Lin, Peng-Peng Zhao, Yuan Mai, Wei Wei, Hua-Cai Zhu, Zhan-Ping Xu

**Affiliations:** a Department of Urinary Surgery, Foshan Hospital of Traditional Chinese Medicine, Foshan, China.

**Keywords:** bioinformatics, bladder cancer (BC), hub genes, robust rank aggregation (RRA), weighted gene co-expression network analysis (WGCNA)

## Abstract

Bladder cancer (BC) is among the most frequent cancers globally. Although substantial efforts have been put to understand its pathogenesis, its underlying molecular mechanisms have not been fully elucidated.

**Methods:**

The robust rank aggregation approach was adopted to integrate 4 eligible bladder urothelial carcinoma microarray datasets from the Gene Expression Omnibus. Differentially expressed gene sets were identified between tumor samples and equivalent healthy samples. We constructed gene co-expression networks using weighted gene co-expression network to explore the alleged relationship between BC clinical characteristics and gene sets, as well as to identify hub genes. We also incorporated the weighted gene co-expression network and robust rank aggregation to screen differentially expressed genes.

**Results:**

CDH11, COL6A3, EDNRA, and SERPINF1 were selected from the key module and validated. Based on the results, significant downregulation of the hub genes occurred during the early stages of BC. Moreover, receiver operating characteristics curves and Kaplan–Meier plots showed that the genes exhibited favorable diagnostic and prognostic value for BC. Based on gene set enrichment analysis for single hub gene, all the genes were closely linked to BC cell proliferation.

**Conclusions:**

These results offer unique insight into the pathogenesis of BC and recognize CDH11, COL6A3, EDNRA, and SERPINF1 as potential biomarkers with diagnostic and prognostic roles in BC.

## 1. Introduction

Bladder cancer (BC), a prevalent urological malignancy, is a global public health concern, and the 9th commonly diagnosed cancer in men, especially in high-income countries.^[1]^ Following a report by Boccardo et al, nearly a quarter BC cases are at first diagnosed as muscle-invasive BC. Moreover, <16% of patients, characterized by non-muscle-invasive BC present with invasive recurrent cancer during treatment, in most cases, within 1 year.^[2]^ As the tumor progresses, BC survival rate declines remarkably. The BC symptoms are usually atypical, without any uniqueness, which poses difficulty in earlier diagnosis.^[3]^ Based on the current understanding, BC diagnosis and surveillance primarily incorporates cystoscopy and urine cytology, however, these approaches are unsatisfactory.^[4]^ Besides, an ideal BC detection technique must be more convenient and rapid. Hence, researchers should urgently uncover more accurate indices for clinical staging, treatment and prognosis of BC.

In this work, we explored 4 independent microarray datasets abstracted from Gene Expression Omnibus web resource (GEO, https://www.ncbi.nlm.nih.gov/geo/) with robust rank aggregation (RRA) to reveal robust differentially expressed genes (DEGs) between BC tissues and matched control. Thereafter, we subjected the DEGs to weighted gene co-expression network analysis (WGCNA) to determine key modules related to clinical parameters. Using the Gene Ontology (GO) functional annotation and Kyoto Encyclopedia of Genes and Genomes (KEGG) pathway analysis, we assessed the potential functions of the genes within the key module. In exploring the biosignatures and targets for BC therapy, we did a range of analyses via mining of sequencing data with high-throughput, retrieved from publicly available databases. Consequently, the present study reported CDH11, COL6A3, EDNRA, and SERPINF1 as potential biomarkers and therapeutic target of BC, and are all linked to the prognosis of individuals with BC.

## 2. Methods

### 2.1. Microarray data

From the GEO web resource (https://www.ncbi.nlm.nih.gov/geo/), we retrieved the GSE13507, GSE7476, GSE65635, as well as GSE37815 gene expression pattern matrix files. The workflow of validation, identification, as well as functional analysis of DEGs are shown in Figure S1, Supplemental Digital Content, http://links.lww.com/MD/I150. The GSE7476 platform is GPL570 (Affymetrix Human Genome U133 Plus 2.0 Array), comprising 9 BC tissues and 3 healthy bladder tissues. The GSE13507 platform is GPL6102 (Illumina human-6 v2.0 expression beadchip), and this dataset had 188 and 68 BC tissues, as well as healthy bladder tissues, respectively. The GSE37815 platform is GPL6102 (Illumina human-6 v2.0 expression beadchip), which harbor 6 and 18 healthy bladder tissues and BC tissues, respectively. The GSE65635 platform is GPL14951 (Illumina HumanHT-12 WG-DASL V4.0 R2 expression beadchip), containing 3 healthy bladder tissues and 9 BC tissues (Table 1).^[5–8]^ In addition, we downloaded the bladder urothelial carcinoma (BLCA) ribonucleic acid sequencing (RNA-seq) and clinical data from The Cancer Genome Atlas (TCGA) web resource (https://cancergenome.nih.gov/) for analysis. The pathological types of BC include: transitional cell papillomas and carcinomas (409 cases), adenomas and adenocarcinomas (1 case), epithelial neoplasms (1case) and squamous cell neoplasms (1case).

**Table 1:** Details of the GEO bladder cancer data.

Dataset ID	Platform	Normal	Tumor	Reference
GSE7476	GPL570	3	9	Mengual et al (2009)^[5]^
GSE13507	GPL6102	68	188	Kim et al (2010), Lee et al (2010)^[6]^
GSE37815	GPL6102	6	18	Kim et al (2013)^[7]^
GSE65635	GPL14951	4	8	Borisov et al (2018)^[8]^

GEO = Gene Expression Omnibus.

### 2.2. Data processing

Employing the GEO website, sequential matrix files of cohorts were retrieved. The R package “limma”^[9]^ was used for data normalization and identify the DEGs. Then, we employed the RRA to integrate the findings of the 4 cohorts to identify DEGs with the highest significance.^[10]^ Genes with a corrected *P* value < .05 and |log fold change (FC)| > 1 were considered as significant DEGs in the RRA analysis.

### 2.3. GO and KEGG pathway analysis

With the Database for Annotation, Visualization and Integrated Discovery (DAVID, https://david.ncifcrf.gov/), important for functional analysis of genes, we performed KEGG pathway enrichment and GO functional analyses, *P* < .05 for statistical significance.

### 2.4. WGCNA analysis of the filtered genes

Herein, 343 DEGs were retrieved following RRA analysis. This aided in obtaining WGCNA with expression data from TCGA. Using the R package “WGCNA,” we uncovered the associated hub genes and clinical traits-related modules.^[11]^ Using the topological overlap measure matrix, transformed through an adjacency matrix, we estimated its network connectivity.^[12]^ Thereafter, we established a hierarchical clustering dendrogram of the topological overlap measure matrix employing the average distance with a value of 20 as the minimum size threshold. This was to group genes with similar expression patterns into distinct gene modules, after which we determined the correlation of different module eigengenes with the clinical features. We evaluated the gene significance (GS) quantifying correlations between individual genes and the module membership (MM) as well as the clinically interesting trait which depicts the association of the module eigengenes with gene expression profiles. Following previous reports, if the GS and MM were highly associated, the highly critical elements in the modules were also strongly linked to the trait.^[13]^ We used the highly correlated module to explore potential function via GO and KEGG analyses and for hub gene screening. Notably, we defined hub genes with: GS > 0.2, and MM > 0.8.

### 2.5. Validation and survival analysis of hub genes

We employed “ggstatsplot” (R packages, https://cran.r-projrct.org/web/packages/ggstatsplot) to verify the levels of expression of hub genes between BC and neighboring healthy tissue sample. Also, we evaluated how they are correlated with clinical traits in TCGA-BLCA dataset. Accordingly, we employed the independent samples *t* test or one-way analysis of variance. To evaluate the diagnosis values of hub genes, we generated receiver operating characteristic (ROC) curves and used “survminer” (R package, https://CRAN.R-project.org/package=survminer) and “survicval” (R package, https://CRAN.R-project.org/package=survival) to calculate for hub genes. For tumor samples within the TCGA-BLCA dataset, we classified them into 2 groups relying on the best-separation cutoff value for each hub gene. After that, we plotted the Kaplan–Meier (KM) survival curves.

The image of immunohistochemistry staining of the selected prognosis-related genes in normal tissue and BC tissues were retrieved from Human Protein Atlas online database (http://www.proteinatlas.org).

### 2.6. Establishment and estimation of multi-gene prognostic signature

Based on the multivariate Cox proportional hazards regression model, 4 optimal prognostic genes were identified. After assembly of expression levels and coefficients for each gene, a linear combination method was applied to get risk scores, which is as follows:


$$\begin{array}{l} Riskscore=\underset{i=1}{\overset{4}{\sum }}\;\beta i*Expi \end{array}$$

where *Exp* is the expression level of each prognostic gene, and *β* is the regression coefficient of it.

A median risk score was used as a cutoff for stratifying patients into high-risk and low-risk groups. As a comparison of the survival differences between above 2 groups, we also used KM survival analysis with log-rank tests.

The prognostic power of the risk score and some clinical parameters, such as age, gender, grade, stage, T-stage, N-stage, M-stage, was assessed by using univariate Cox proportional hazards regression analysis. Moreover, we assessed whether the risk scores could be independent prognostic factors based on levels of risk using multivariate Cox proportional hazards regression analysis for PCa patients. Other clinical parameters with statistically significant difference (*P* < .05) in univariate Cox proportional hazards regression were also incorporated in the analysis.

### 2.7. Oncomine database

Herein, we retrieved transcriptional expression profiles of CDH11, COL6A3, EDNRA, and SERPINF1 in BC patients using the Oncomine web resource (https://www.oncomine.org).^[14]^ To compare the differences in transcriptional expression, we employed Student *t* test with fold change and cutoff of *P* value as follows: data type: messenger ribonucleic acid (mRNA), *P* value = .01, gene rank = 10%, fold change = 1.5.

### 2.8. Tumor Immune Estimation Resource (TIMER)

TIMER (https://cistrome.shinyapps.io/timer/) offers a web interface, which is user friendly, important for dynamic analysis of the associations of immune infiltrates with gene expression.^[15]^ Using the gene module, we validated the association between immune infiltration and genes. We then generated scatterplots, depicting statistical significance and Spearman correlation.

### 2.9. Data processing of gene set enrichment analysis (GSEA)

Using the R package “clusterprofiler,”^[16]^ we conducted a GSEA analysis of hub genes using TCGA-BLCA RNA-dataset. For each hub gene, we determined the median expression by classifying 414 BLCA samples into high and low expression groups. We considered *P* < .01 to be statistically significant. For the reference gene set, we used “h.all.v7.1.symbols.gmt,” abstracted from the Molecular Signature Database (MSigDB, http://software.broadinstitute.org/gsea/msigdb/index.jsp).

### 2.10. Statistical analysis

The results were given as means ± standard deviation of independent experiments. *P* values were calculated using SPSS v. 24.0 software with unpaired, 2-tailed Student *t* test or which indicated with one-way analysis of variance followed by Turkey test. *P* values of <.05 were considered to indicate statistical significance. *P < .05, **P < .01, and ***P < .001.

## 3. Results

### 3.1. Identifying robust DEGs via the RRA method

**Figure 1.**
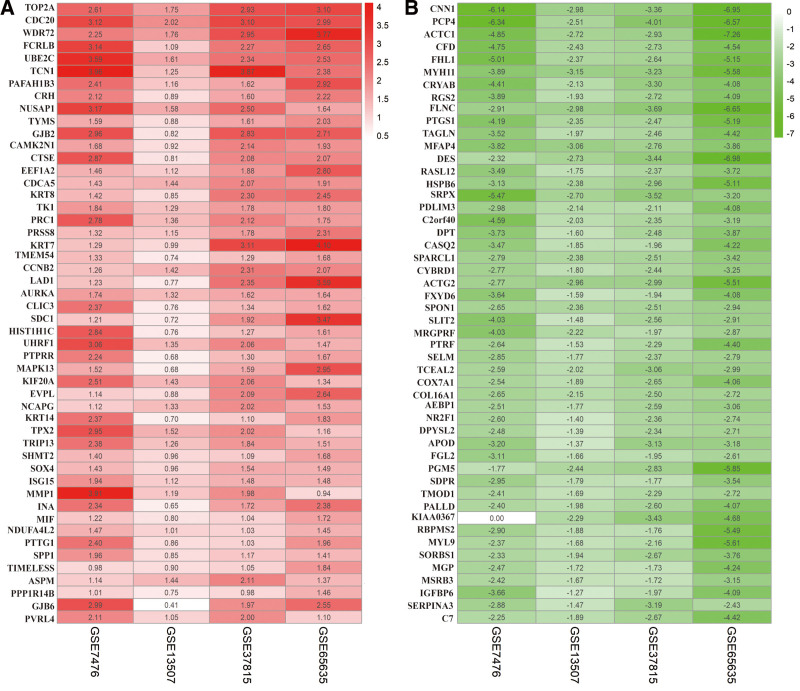
Identifying robust DEGs via RRA analysis. (A) A heatmap highlighting the top 50 upregulated genes and (B) 50 downregulated genes based on the *P* value. Every row denotes the gene name, whereas each column shows the GEO IDs. Red denotes upregulation, whereas green denotes downregulation. DEGs = differentially expressed genes, GEO = Gene Expression Omnibus, RRA = robust rank aggregation.

Using the selection criteria, 4 independently eligible BLCA datasets were enrolled for subsequent RRA analysis. A series of clinical traits, including GEO accession ID, platform ID, as well as the number of genes for each platform are displayed in Table 1.^[5–8]^ Based on RRA analysis data, we identified 111 upregulated and 232 downregulated remarkable DEGs (Supplementary file 1, Supplemental Digital Content, http://links.lww.com/MD/I153, which illustrates 343 remarkable DEGs). Besides, the top 50 upregulated, as well as downregulated DEGs are depicted in the heatmap (Fig. 1).

### 3.2. Functional enrichment analysis of DEGs

**Figure 2.**
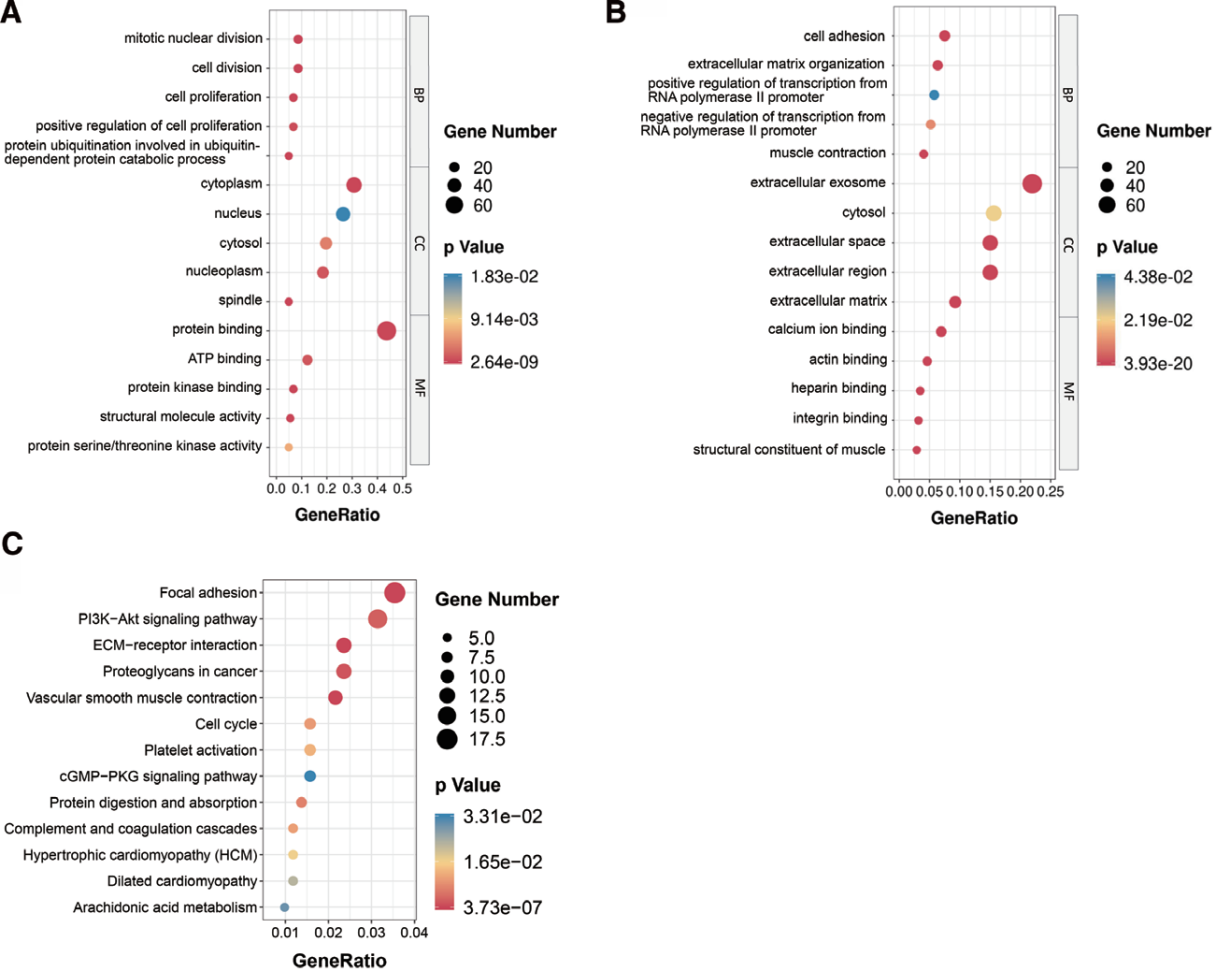
Distribution of integrated DEGs in bladder cancer for different GO-enriched functions and KEGG pathway enrichment analysis. (A) Upregulated DEGs for GO-enriched functions. (B) Downregulated DEGs for GO-enriched functions. (C) KEGG pathway enrichment analysis. DEGs = differentially expressed genes, GO = Gene Ontology, KEGG = Kyoto Encyclopedia of Genes and Genomes.

The biologically functioning DEGs were revealed via the GO and KEGG functional enrichment analysis using DAVID. We considered the results significant only if *P* < .05, we have highlighted the 3 categories of the GO results in Figure 2A and B. Results on the upregulated and downregulated DEGs in top 15 findings derived from the GO enrichment analysis are depicted in Table 2 and Table 3. Of note, the upregulated genes were highly enriched in protein binding (ontology: MF), nuclear division during mitosis (ontology: BP), and cytoplasm (ontology: CC). Besides, the downregulated genes were highly abundant in, extracellular exosome (ontology: CC), and binding of calcium ions (ontology: MF) and cell adhesion (ontology: BP). As to KEGG pathway analysis, extracellular matrix (ECM)-receptor interaction, focal adhesion, P13K-Akt signaling cascade, proteoglycans in cancer, as well as vascular smooth muscle contraction, were mostly associated with these genes (Fig. 2C).

**Table 2:** Top 15 GO enrichment terms linked to the upregulated genes.

Category	Term	Count	*P* value
BP	Mitotic nuclear division	14	2.64E–09
BP	Cell division	14	1.56E–07
BP	Cell proliferation	11	6.49E–05
BP	Positive regulation of cell proliferation	11	4.58E–04
BP	Protein ubiquitination involved in ubiquitin-dependent protein catabolic process	8	3.58E–05
CC	Cytoplasm	50	8.86E–05
CC	Nucleus	43	0.018272
CC	Cytosol	32	0.003596
CC	Nucleoplasm	30	9.69E–04
CC	Spindle	8	6.76E–06
MF	Protein binding	71	1.15E–04
MF	ATP binding	20	9.88E–04
MF	Protein kinase binding	11	7.14E–05
MF	Structural molecule activity	9	1.02E–04
MF	Protein serine/threonine kinase activity	8	0.006637

ATP = adenosine triphosphate, BP = biological process, CC = cellular component, GO = Gene Ontology, MF = molecular function.

**Table 3:** Top 15 GO enrichment terms associated with the downregulated genes.

Category	Term	Count	*P* value
BP	Cell adhesion	26	9.09E–10
BP	Extracellular matrix organization	22	6.93E–14
BP	Positive regulation of transcription from RNA polymerase II promoter	20	0.04382
BP	Negative regulation of transcription from RNA polymerase II promoter	18	0.010419
BP	Muscle contraction	14	9.69E–10
CC	Extracellular exosome	76	1.22E–11
CC	Cytosol	54	0.022837
CC	Extracellular space	52	2.83E–13
CC	Extracellular region	52	2.08E–10
CC	Extracellular matrix	32	3.93E–20
MF	Calcium ion binding	24	2.90E–05
MF	Actin binding	16	2.03E–06
MF	Heparin binding	12	4.99E–06
MF	Integrin binding	11	7.08E–07
MF	Structural constituent of muscle	10	1.80E–09

BP = biological process, CC = cellular component, GO = Gene Ontology, MF = molecular function, RNA = ribonucleic acid.

### 3.3. WCGNA analysis and modules significance calculation

**Figure 3.**
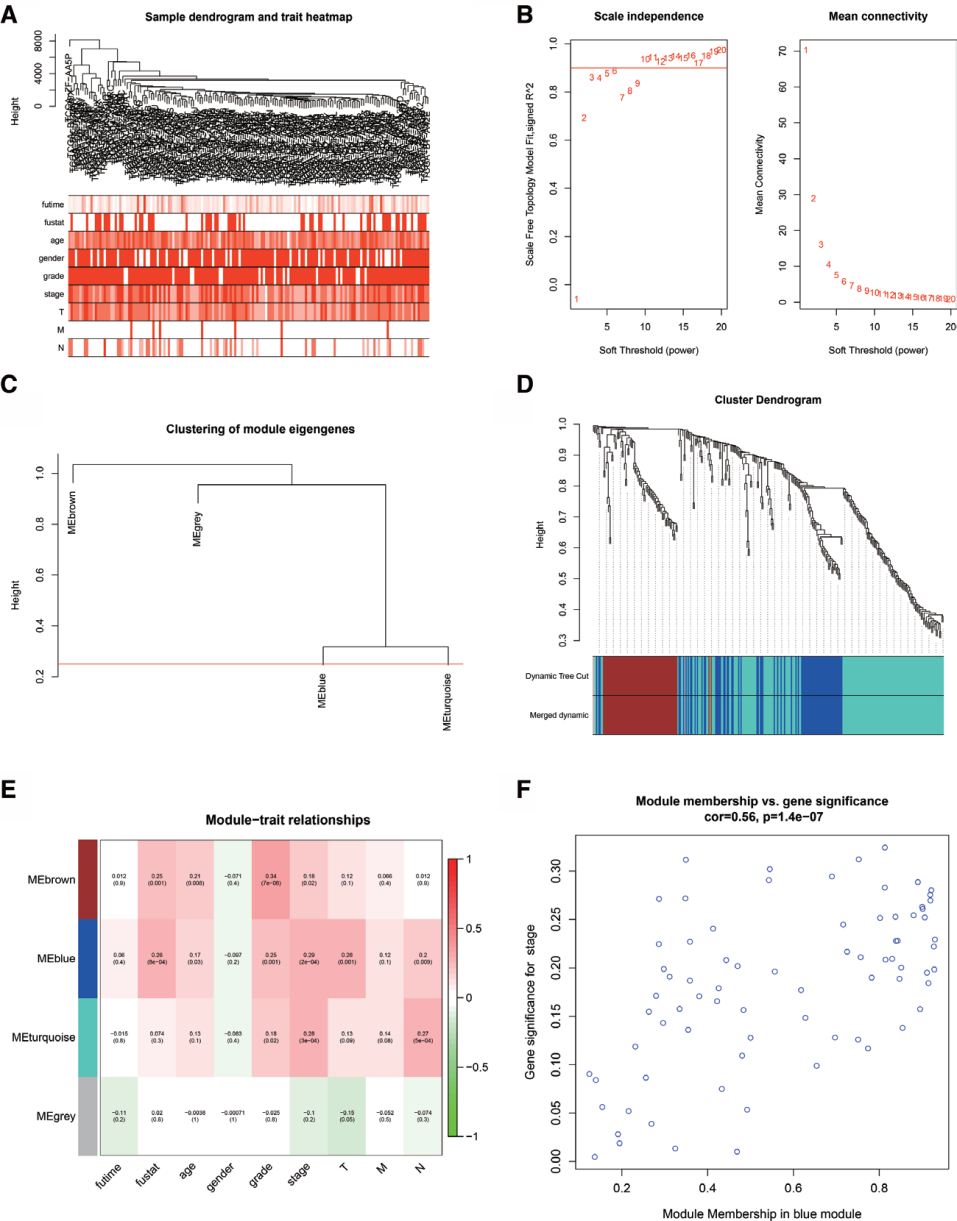
Identifying the key modules associated with clinical features in the TCGA-PRAD cohort using WGCNA. (A) Clustering dendrograms of genes, based on the TCGA-BLCA RNA-seq data of robust DEGs from RRA analysis. There is a positive variation of color intensity with age, grade and pathological stage. (B) Scale-free fit index (left), as well as the mean connectivity (right) analyses for various soft-thresholding powers. (C) Clustering of module eigengenes. The red line denotes the cut height (0.25). (D) Dendrogram of all DEGs clustered based on a dissimilarity measure (1-TOM). (E) Heatmap of the correlation of module eigengenes with clinical features of BLCA. Each cell shows the correlation coefficient and *P* value. (F) Scatter plot of module eigengenes are denoted in the blue module. BLCA = bladder urothelial carcinoma, DEGs = differentially expressed genes, RNA-seq = ribonucleic acid sequencing, RRA = robust rank aggregation, TCGA- PRAD = The Cancer Genome Atlas Prostate Adenocarcinoma, TOM = topological overlap measure, WGCNA = weighted gene co-expression network analysis.

**Figure 4.**
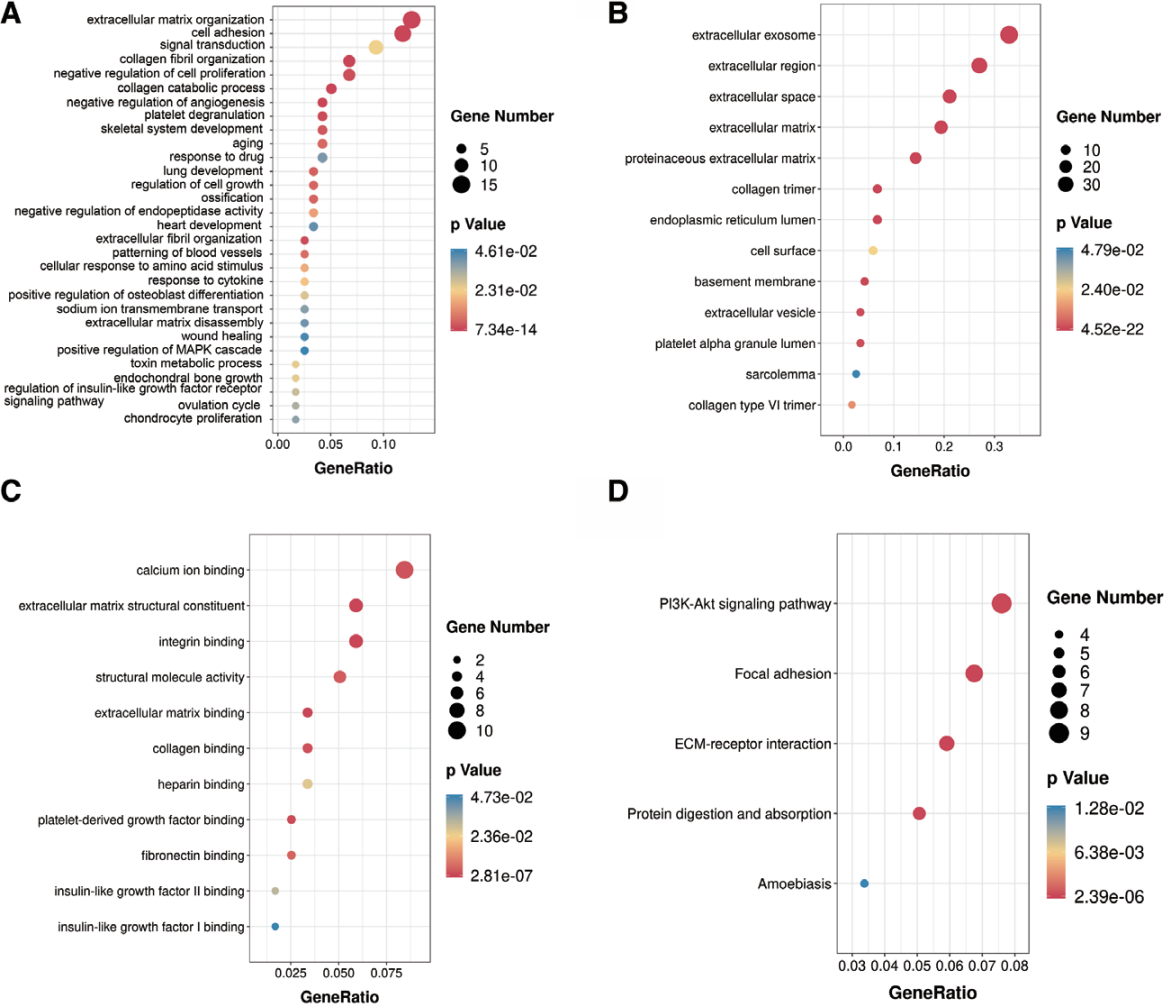
The functional annotation of the WGCNA module highly correlated with clinical traits. (A) Biological process GO terms for genes in the blue module. (B) Cellular component GO terms for genes in the blue module. (C) Molecular function GO term for genes in the blue module. (D) KEGG analysis for genes in the blue module. GO = Gene Ontology, KEGG = Kyoto Encyclopedia of Genes and Genomes, WGCNA = weighted gene co-expression network analysis.

To reveal the key modules highly related to the clinical characteristics of BC, we analyzed the WGCNA on the TCGA-BLCA cohort by integrating the DEGs retrieved from the RRA analysis (Fig. 3). Clinical information of BC sample from TCGA, including stage, age, grade, and TNM classification were retrieved (Fig. 3A). We set the soft-thresholding power at 6 (scale free *R*^2^ = 0.9) and cut height as 0.25. Consequently, 4 modules were identified (Fig. 3B–D). Based on the heatmap showing module-trait correlations, the blue module shows the highest correlation with clinical symptoms (Fig. 3E), particularly the stage (*R* = 0.56, *P* < .001) (Fig. 3F). The blue module had 67 genes (see Supplementary file 2, Supplemental Digital Content, http://links.lww.com/MD/I154, which illustrates the blue module of WGCNA). We set the MM > 0.8 and GS > 0.2 then identified 19 hub genes from the blue module: EDNRA, SERPINF1, COLEC12, FBLN5, DDR2, SFRP2, OLFML3, AEBP1, DCN, CDH11, TIMP2, LUM, DPT, COL6A3, COL16A1, EMILINN1, SPON1, OLFML1, and CRISPLD2. Through GO and KEGG analyses, we uncovered the prospective biological roles of the genes in the blue module. The highest remarkable GO terms for biological process, molecular function, and cellular component, as well as KEGG pathways, are depicted in (Fig. 4A–D). Following this evaluation, genes within the blue modules were primarily linked to signal transduction, cell adhesion, and ECM organization.

### 3.4. Survival analysis and significant gene identification

**Figure 5.**
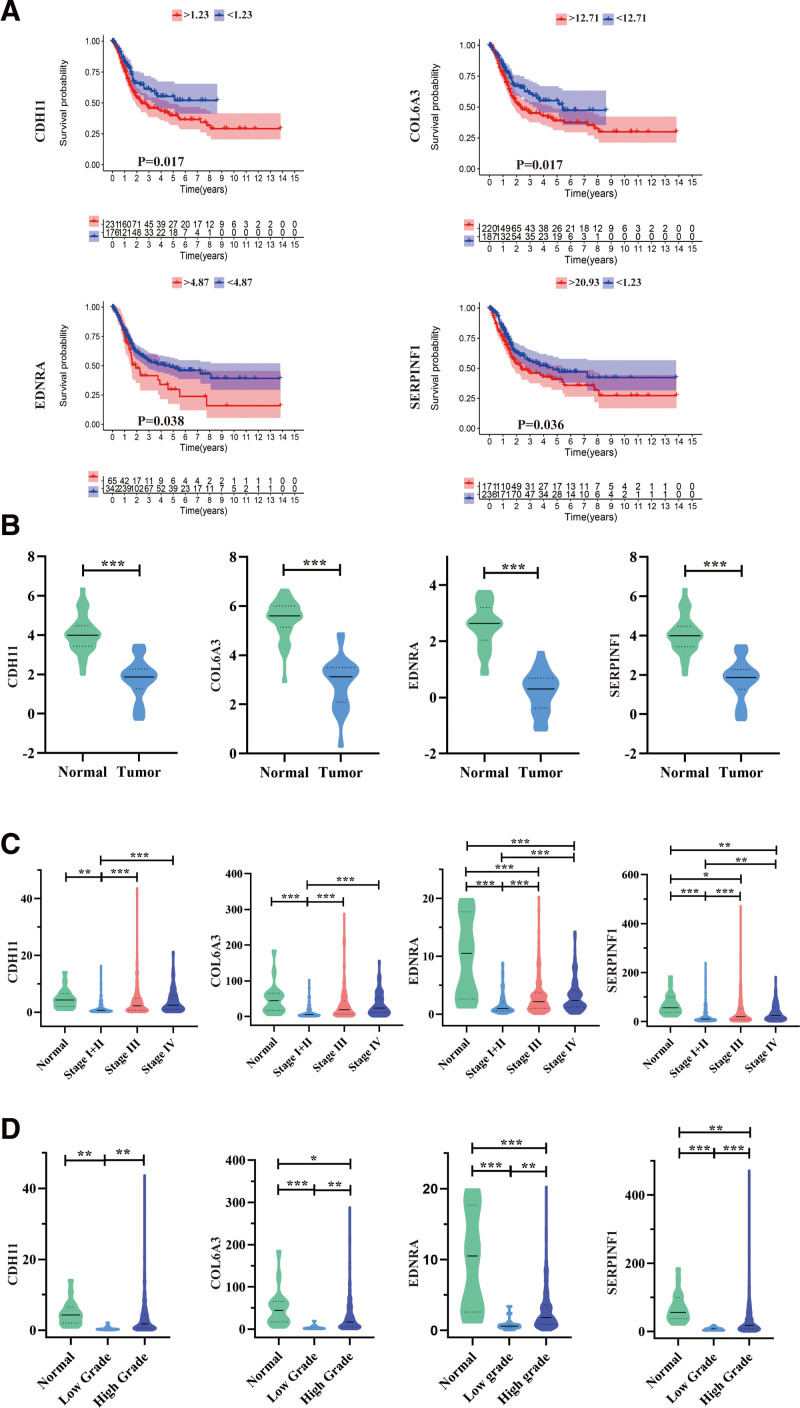
Survival plot and transcriptional expression of hub genes in bladder tumor samples and neighboring healthy tissues. (A) Association between CDH11, COL6A3, EDNRA, and SERPINF1 expression and disease-free survival time in the TCGA-PRAD cohort. The red line shows samples with highly expressed genes (above best-separation value), and the blue line indicates the samples with lowly expressed genes (below best-separation value). (B) CDH11, COL6A3, EDNRA, and SERPINF1 gene expression differences between BC and neighboring healthy tissues from the Oncomine dataset. (C) Transcriptional level of CDH11, COL6A3, EDNRA, and SERPINF1 expression in BC samples with different stages from the TCGA-BLCA cohort. (D) Transcriptional level of CDH11, COL6A3, EDNRA, and SERPINF1 expression in BC samples with different grades from the TCGA-BLCA dataset. *P < .05, **P < .01, and ***P < .001. BC = bladder cancer, BLCA = bladder urothelial carcinoma, TCGA- PRAD = The Cancer Genome Atlas Prostate Adenocarcinoma.

We assessed whether the 19 hub genes in BC were clinically relevant. To achieve this, correlation assessment of the hub genes with prognosis outcome of BC patients in TCGA-BLCA data sets was performed. By optimizing the cutoff values for hub gene analysis, CDH11, COL6A3, EDNRA, and SERPINF1 were highly expressed and were associated with poor prognosis (Fig. 5A and Figure S2, Supplemental Digital Content, http://links.lww.com/MD/I151, which analyses all hub genes’ survival in the WGCNA blue module). Furthermore, ROC curves demonstrated that they had high diagnostic potential as BC biosignatures (Figure S3, Supplemental Digital Content, http://links.lww.com/MD/I152; CDH11 area under the ROC curve (AUC): 0.699, COL6A3 AUC: 0.697, EDNRA AUC: 0.833, SERPINF1 AUC: 0.804), suggesting the potential use of the genes as indicators in monitoring prognosis.

### 3.5. Differential expression of CDH11, COL6A3, EDNRA, and SERPINF1

**Figure 6.**
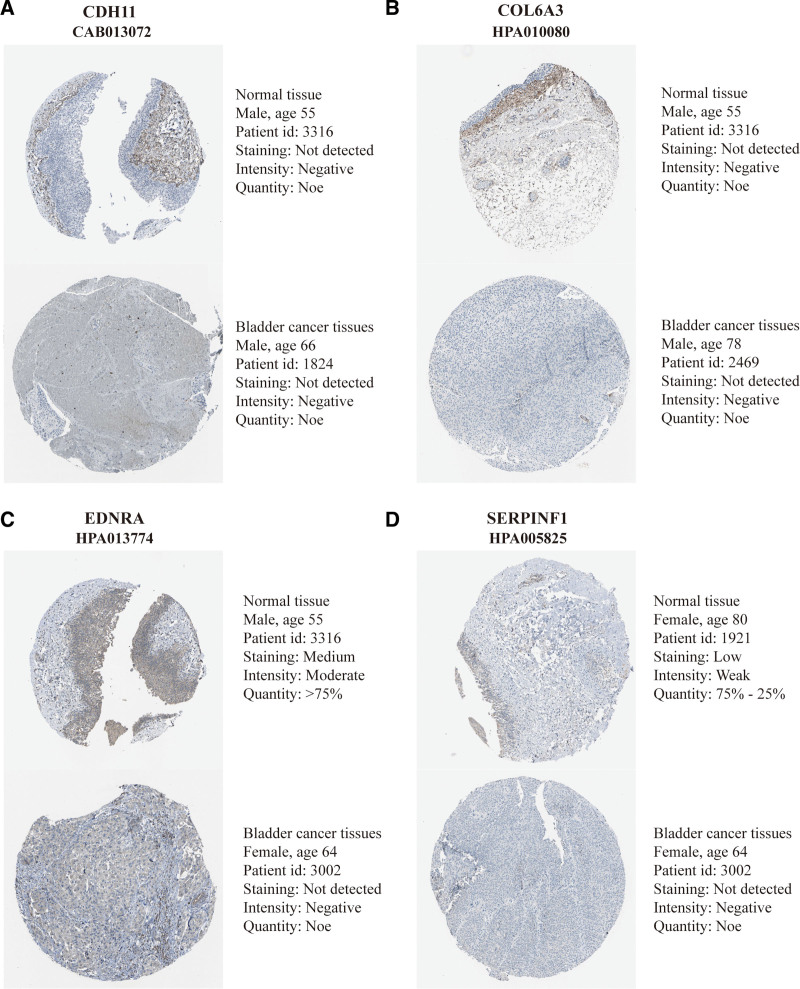
Analysis of protein expression of 4 hub genes. The protein expression of (A) CDH11, (B) COL6A3, (C) EDNRA, and (D) SERPINF1 in normal and tumor bladder tissue was obtained from the Human Protein Atlas.

We compared the mRNA expression of CDH11, COL6A3, EDNRA, and SERPINF1 between bladder tumor and neighboring healthy tissues, respectively. This was based on data for RNA-seq obtained from the Oncomine and TCGA databases. Notably, the transcriptional levels of CDH11, COL6A3, EDNRA, and SERPINF1 expressions were lowly expressed in BC tissues in comparison to healthy tissues (Fig. 5B). Moreover, Human Protein Atlas database was used to validate the protein expression of the 4 genes. However, no difference was found for CDH11 and COL6A3 protein expression (Fig. 6). Besides, there was a significant correlation of CDH11 mRNA expression and BC samples with a mild clinical stage (Fig. 5C), whereas the lowest CDH11 mRNA expression was reported stage I + Ⅱ. Similarly, we evaluated the association of CDH11 mRNA expression with different pathological grade, whereby it was revealed that mRNA expression of CDH11 is significantly correlated with lower pathological grades (Fig. 5D). Additionally, mRNA levels of COL6A3, EDNRA, and SERPINF1 were lower in BC tissues (Fig. 5B). COL6A3, EDNRA, and SERPINF1 mRNA expression in BLCA sample were significantly correlated with mild clinical staging, whereas the lowest COL6A3, EDNRA, and SERPINF1 mRNA expression were detected in stage Ⅰ + Ⅱ (Fig. 5C). Moreover, mRNA expression levels of COL6A3, EDNRA, and SERPINF1 were related to lower clinicopathological grading (Fig. 5D). Collectively, we demonstrated that the expressions of CDH11, COL6A3, EDNRA, and SERPINF1 were lower in BC tissues compared to healthy tissues. Thus, the hub gene CDH11, COL6A3, EDNRA, and SERPINF1 could play a pivotal role in BC progression. Overall, low expression of CDH11, COL6A3, EDNRA, and SERPINF1 mRNA is significantly associated with mild clinical-pathological parameters in BC patients and is significantly lowered in the early disease stages. This may be vital in the early BC diagnosis.

### 3.6. Identification and estimation of a 4-gene prognostic signature

**Figure 7.**
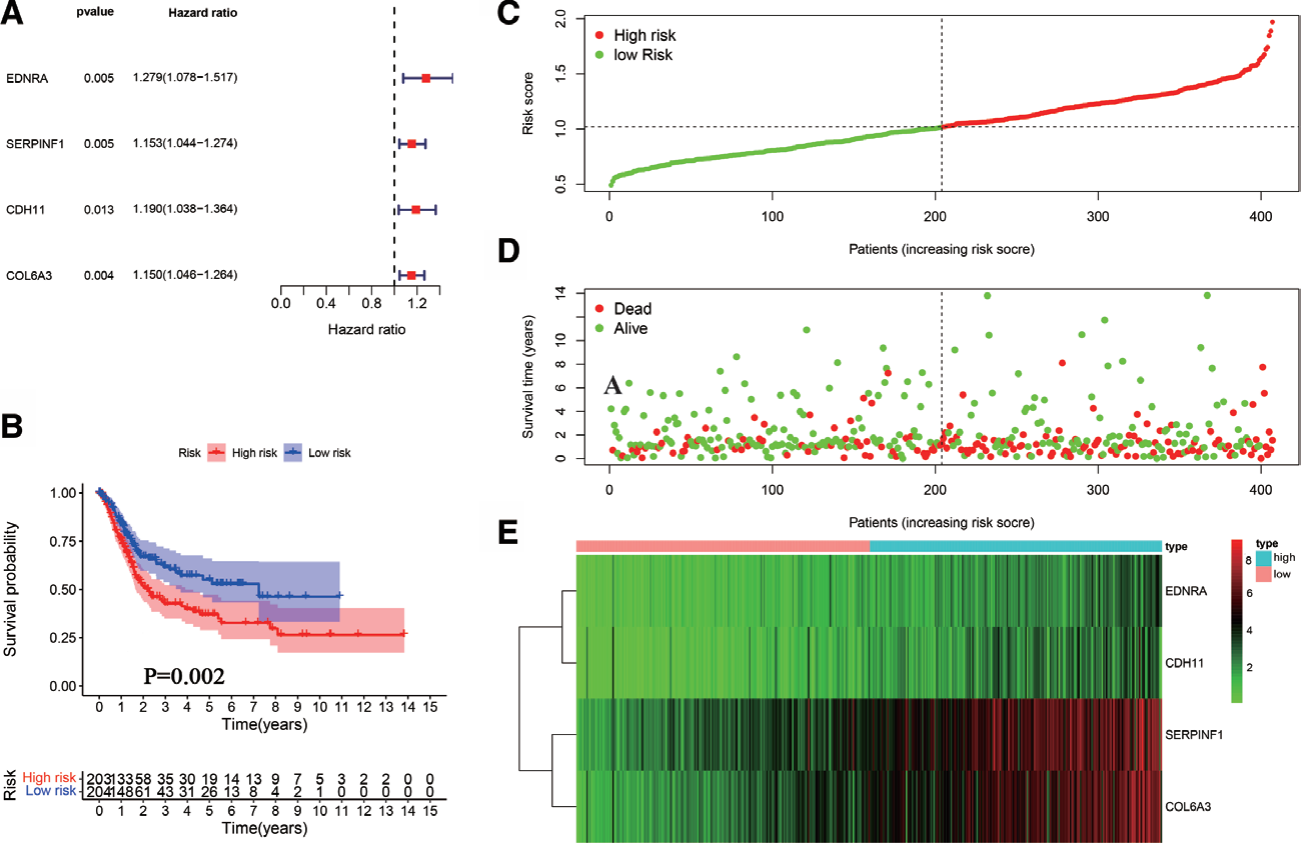
Prognostic analysis of 4 gene signature. The dotted line was used to represent the median risk score, the patients were divided into low- and high-risk groups. (A) Multivariate Cox regression analysis of 4 prognostic gene. (B) KM survival analysis of the 4 gene signature. (C) The curve of risk score. (D) Survival status of the patients. (E) Heatmap of the expression profiles of the 4 prognostic genes in low- and high-risk group. KM = Kaplan–Meier.

**Figure 8.**
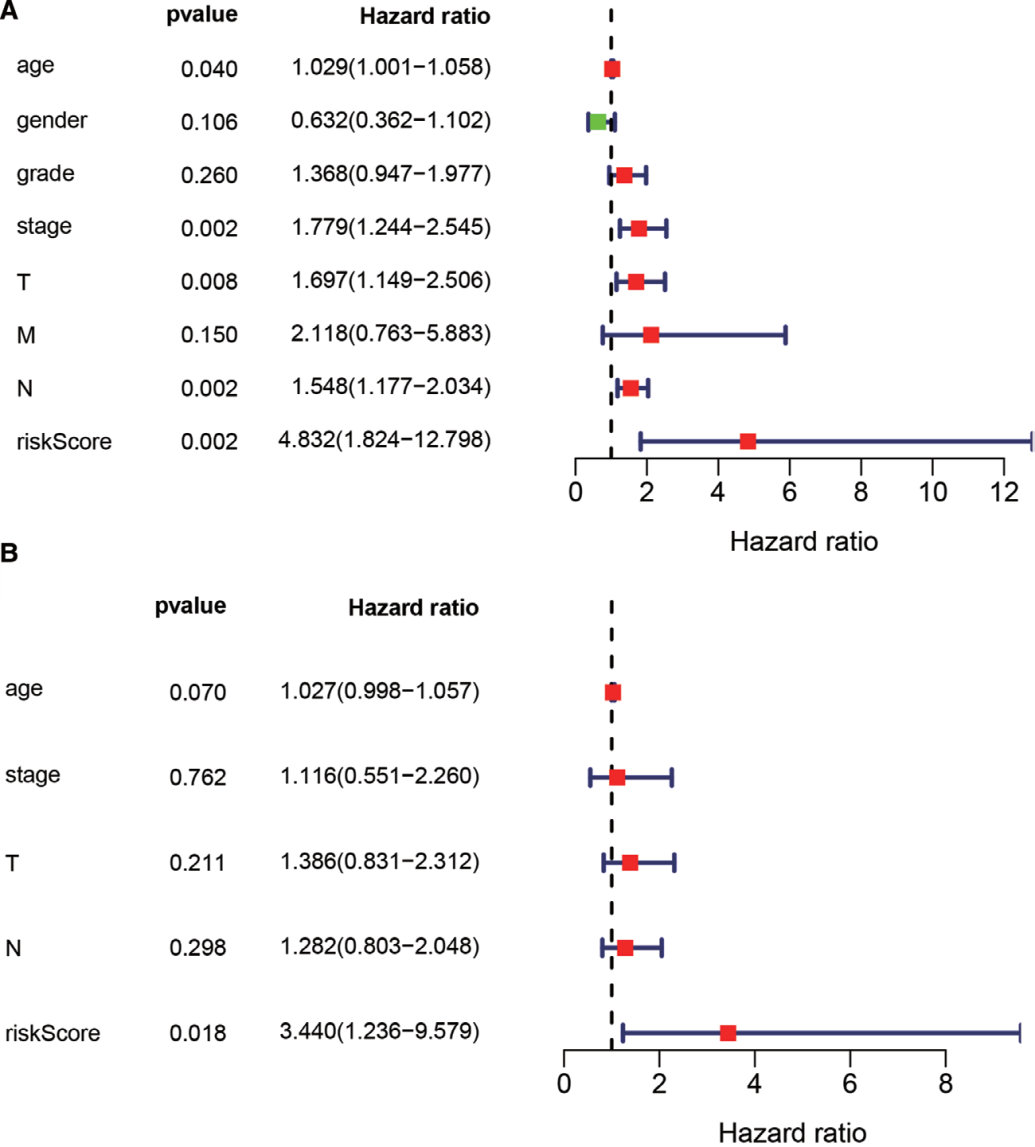
Identifying the independent prognostic parameters of PCa. (A) Forrest plot of univariate Cox regression analysis in PCa. (B) Forrest plot of multivariate Cox regression analysis in PCa. PCa = .

A multivariate Cox proportional hazards regression analysis of 4 prognostic genes was conducted to determine the prognostic significance of each gene for PCa patients (Fig. 7A). Thus, we constructed a gene-based risk score based on their Cox coefficients: risk score = −0.191090924*Exp_(CDH11) _+ 0.141406166*Exp_(COL6A3_) + 0.099154851*Exp_(EDNRA) _+ 0.101448872*Exp_(SERPINF1)_. In the next step, the risk score of every patient was calculated, based on which we obtained the median cutoff point using the R package “survminer” and divided the patients into 2 groups, high risk group (n = 203) and low risk group (n = 204) (Fig. 7C). All patients’ survival states are shown in Figure 7D, while the heatmap of 4 prognostic genes can be seen in Figure 7E. As indicated by the KM survival curves (Fig. 7B), high-risk patients had a worse overall survival than low-risk patients. The univariate and multivariate Cox regression analysis demonstrated that other clinical parameters, such as age, gender, grade, stage and TNM stage were not independent risk factors of the overall survival, except for the 4 genes prognostic signature (Fig. 8).

### 3.7. Association of hub genes’ expression with tumor-infiltrating immune cells

**Figure 9.**
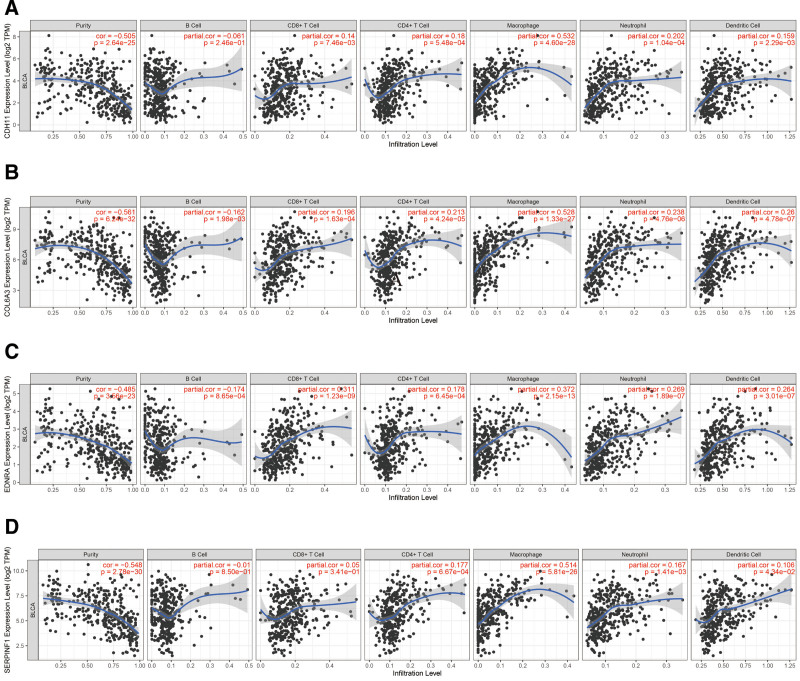
Integrative analysis of the established hub immune biosignature with tumor-infiltrating immune cells. (A) CDH11. (B) COL6A3. (C) EDNRA. (D) SERPINF1. *P* < .05 show statistically significant difference, whereas each dot denotes a sample in the TCGA-BLCA cohort. TCGA-BLCA = The Cancer Genome Atlas Bladder Urothelial Carcinoma.

Referring to the critical roles of invading immune cells within the tumor microenvironment, we comprehensively analyzed immune signatures plus immune infiltrates. From the TIMER web resource, the association between CDH11, COL6A3, EDNRA, and SERPINF1 immune signatures and tumor purity or numerous vital immune cells was revealed. CDH11, COL6A3, EDNRA, and SERPINF1 were all negatively correlated with tumor purity. The correlations (Cor > 0.5 and *P* < .05) were considered to be the strongest correlated. Although it was observed no or weak correlations of these genes with infiltration of CD8^ + ^T cells, dendritic cells, CD4^ + ^T cells, B cells, and neutrophils, CDH11, COL6A3 and SERPINF1 were significantly associated with macrophages (Fig. 9).

### 3.8. GSEA analysis

**Figure 10.**
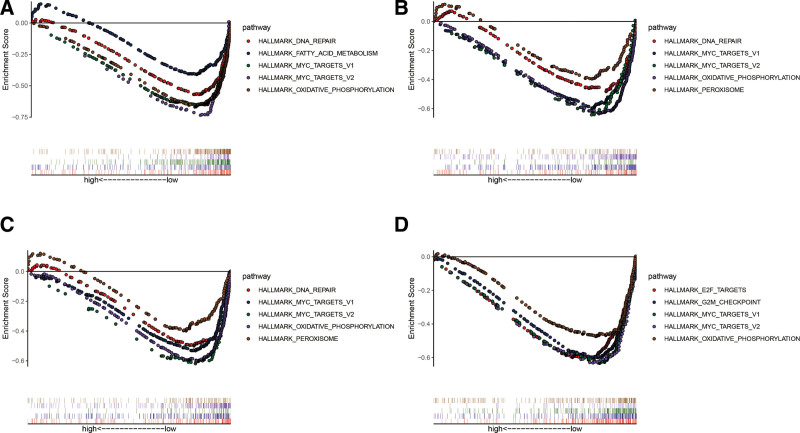
Gene set enrichment analysis (GSEA) of hub genes in the TCGA-BLCA dataset. (A–D) Top 5 gene sets (according to GSEA enrichment score) abundant in the high-expression group of single hub genes. (A) CDH11, (B) COL6A3, (C) EDNRA, and (D) SERPINF1. TCGA-BLCA = The Cancer Genome Atlas Bladder Urothelial Carcinoma.

To assess the potential roles of CDH11, COL6A3, EDNRA, and SERPINF1 in BC, GSEA was conducted for hallmark analysis of the genes on the TCGA-BLCA RNA-seq data. Genes in low expression CDH11, COL6A3, EDNRA, and SERPINF1 groups were enriched in “MYC-TARGETS-V2” “MYC-TARGETS-V1,” and “OXIDATIVE-PHOSPHORYLATION” pathways (Fig. 10). Meanwhile, the “DNA-REPAIR” gene set was abundant in low-expression groups of CDH11, COL6A3 and EDNRA, and “PEROXISOME” was enriched in the COL6A3 and EDNRA low-expression groups.

## 4. Discussion

BC, being the most prevalent malignant tumors of the genitourinary system has in recent years, shown an increasing incidence. More importantly, identifying the prognostic, as well as predictive biosignatures for BC is vital because BC is a diverse disease with unpredictable clinical endpoints.^[3]^ A wealth of studies have shown that progression of BC is attributed to the accumulation of cellular and molecular aberrations, such as transcriptomic, miRNA, epigenetic, metabolomic and proteomic abnormalities.^[17–20]^ Following the multiple “omics” research that purposed to reveal diagnostic biomarkers for early BC detection, both the heterogeneity and the potential commonalities at the molecular level were highlighted in different BC stages. Of note, there is evidence on BC molecular heterogeneity, associated with several changes at genetic and protein levels. Therefore, a bunch of comprehensively-selected candidates could be representative of these tumors. Several assessments employing microarray and RNA-seq data have been performed to uncover novel therapeutic targets and biomarkers for BC; however, inconsistencies exist on the DEGs detected in various studies.^[21,22]^ Of interest, we present the first report to the use of RRA-WGCNA to explore novel hub genes related to BLCA.

In the present work, unlike a single genetic or cohort study, we incorporated 4 qualified BLCA datasets from GEO into the RRA technique, after which several robust DEGs were identified. In total, 343 DEGs were revealed, including 111 upregulated and 232 downregulated genes. Then, we conducted GO based on DAVID, which demonstrated that the DEGs were mainly abundant in cell division, mitotic nuclear division, cell proliferation, protein kinase binding and protein serine/threonine kinase activity. Based on these observations, we confirmed their role in BC development.^[3,23]^ Additionally, enrichment of the DEGs in some KEGG pathways, for instance, ECM-receptor interaction and focal adhesion implicate that they are essential in the pathogenesis of BC. Following GO and KEGG analysis findings, we proposed that the DEGs have a close association with the development of BC.

Moreover, upon constructing the co-expression network, as well as identifying the hub genes via WGCNA, we revealed that genes within the co-expression module which are highly associated with clinical features of BLCA samples in TGCA (blue module) were enriched in: signal transduction, cell adhesion, P13K-Akt signaling pathway as well as ECM-receptor interaction by GO and KEGG analyses (Fig. 4). After filtering for GS and MM value, 19 hub genes (EDNRA, SERPINF1, COLEC12, FBLN5, DDR2, SFRP2, OLFML3, AEBP1, DCN, CDH11, TIMP2, LUM, DPT, COL6A3, COL16A1, EMILINN1, SPON1, OLFML1, and CRISPLD2) were eventually obtained. Notably, most of them could exert essential functions in BC pathogenesis.^[24–27]^ Moreover, after performing survival analysis, CDH11, COL6A3, EDNRA, and SERPINF1 were revealed as the only 4 outstanding genes (Fig. 5A).

CDH11 (cadherin-11), which is a cadherin superfamily member, a group of intercellular adhesion molecules dependent on calcium, which are critical for adhesion, proliferation and invasion of cells.^[28,29]^ The expression of CDH11 has been correlated to numerous pathologic processes, including fibrosis and inflammation, which is essential as it progresses from chronic inflammation to cancer.^[30,31]^ Besides, CDH11 has been implicated in breast, prostate, colorectal cancer metastases.^[32–34]^ However, based on recent studies, CDH11 functions as a gene that suppresses tumors, upon CDH11 inactivation, which is linked to the malignant characteristics of different human tumors.^[35,36]^ However, the association of CDH11 with BC is yet to be fully elucidated. COL6A3 (collagen VI α 3), a protein of the ECM, is present in a majority of connective tissues, such as skin, muscle, vessels, and tendons.^[37]^ Based on recent understanding, numerous studies have outlined the critical function of COL6A3 in the prognosis and diagnosis of prostate, lung, and colorectal cancers.^[38–40]^ Besides the above findings, the use of COL6A3 to diagnose and prognose BC is still elusive. EDNRA is a G-protein coupled endothelins receptor which is expressed on vascular smooth-muscle cells as well as on neuronal cells, kidney, and heart.^[41]^ Notably, the potential functional effects of EDNRA in metastasis and cancer progression remain unclear. SERPINF1, also known as pigment epithelium-derived factor, is secreted as a protein with multiple functions. It impedes metastasis and angiogenesis, promotes tumor cell differentiation and apoptosis, and activates cellular immunity in fighting breast cancer, cervical cancer, and melanoma.^[42–44]^ Of note, SERPINF1 promotes vascular microenvironment maturation and regression of immature blood vessels.^[45]^ Some reports show that SERPINF1 potentially impede the migration and proliferation simultaneously, which is induced via the vascular endothelial growth factor.^[46]^ Consequently, it inhibits angiogenesis through the interaction with specific cell surface receptors,^[46]^ though its actual role in BC progression is unclear.

The mRNA expression of these DEGs were verified in normal tissues and BC tissues from the Oncomine and TCGA databases. We found via immunohistochemical analysis that mRNA expression was not completely consistent with the protein expression. For 2 genes, protein expression was not differential between cancer and normal tissues. The possible reason is that the process of mRNA translation into protein may be regulated by noncoding RNA, including microRNA, circular RNA, and long non-coding RNA,^[47–49]^ or may be subject to epigenetic modifications, such as m6A modification.^[50]^

In this study, we found that the mRNA expression of hub genes in cancer tissues was lower than that in normal tissues, but the genes were upregulated in higher tumor grades (Fig. 5B–D). For example, the mRNA of EDNRA was overexpressed in samples with higher T stages. The possible explanation is that downregulation of genes is the initiating factor of BC, but the expression level gradually increases during the disease progression or as a result of other regulation of oncogene expression. Moreover, ROC curves demonstrated that all the 4 genes, when adopted as biomarkers could distinguish tumors from healthy bladder tissue in a more sensitive and accurate manner. It is worth noting that all these genes are prospective candidates as prognosis predictors as well as therapeutic targets. Then, in the univariate and multivariate Cox regression analyses, the 4-gene signature was found to be an independent factor in the prognosis evaluation (Fig. 8).

For the hub genes, we further explored their biological functions by inferring to the TIMER dataset and GSEA. It was noted that the expression of CDH11, COL6A3, EDNRA, and SERPINF1 were negatively associated with tumor purity. However, we did not find any or weak relationships for hub genes and invading immune cells except for macrophages in BC tissues. Based on TIMER results, we suggested that CDH11, COL6A3 and SERPINF1 may exhibit their macrophage-associated functions (Fig. 9). Recent studies also revealed that macrophages enhance the tumorigenesis and increase aggressive clinical manifestations of BC.^[51,52]^ GSEA showed that significant pathways for CDH11, COL6A3, EDNRA, and SERPINF1 include “MYC-TARGETS-V1,” “MYC-TARGETS-V2” and “OXIDATIVE-PHOSPHORYLATION” (Fig. 10). Of note, all the gene sets with the highest enrichment scores had a close association with tumor proliferation.^[53–55]^

## 5. Conclusion

In a nutshell, the present study integrated RRA, WGCNA with other bioinformatics tools to identify and characterize numerous robust DEGs and significant gene modules in BC. Of note, 4 hub genes (CDH11, COL6A3, EDNRA, and SERPINF1) were strongly downregulated in BC tissues, which may be vital in uncovering the underlying mechanisms related to BC progression and provide more insights into its molecular pathogenesis in addition to defects in the signaling pathways of hub genes associated with the BC. The hub genes in this study were extracted by bioinformatics techniques, and further experimental studies are needed to determine whether they can regulate the biological functions of BC cells. The specific mechanism also must be proved and explored in more detail.

## Acknowledgments

We wish to acknowledge that information analyzed in this work have been retrieved from The Cancer Genome Atlas database (TCGA), Gene Expression Omnibus (GEO), the Oncomine database and Human Protein Atlas online database. We highly appreciate having been granted the access.

## Author contributions

**Conceptualization:** Fu Feng (equal), Zhan-Ping Xu (equal).

**Data curation:** Jian-Hua Huang (equal), Fu-Xiang Lin (equal), Peng-Peng Zhao (equal), Wei Wei (equal), Hua-Cai Zhu (equal).

**Formal analysis:** Yu-Xiang Zhong (equal), Fu-Xiang Lin (equal), Peng-Peng Zhao (equal), Yuan Mai (equal).

**Funding acquisition:** Zhan-Ping Xu (lead).

**Methodology:** Yu-Xiang Zhong (equal), Fu-Xiang Lin (equal), Peng-Peng Zhao (equal), Wei Wei (equal), Hua-Cai Zhu (equal).

**Project administration:** Fu Feng (equal), Zhan-Ping Xu (equal).

**Software:** Fu Feng (equal), Jian-Hua Huang (equal), Yuan Mai (equal).

**Supervision:** Zhan-Ping Xu (lead).

**Visualization:** Jian-Hua Huang (equal).

**Writing – original draft:** Fu Feng (lead), Yu-Xiang Zhong (equal), Yuan Mai (equal), Wei Wei (equal), Hua-Cai Zhu (equal).

**Writing – review & editing:** Fu Feng (equal), Zhan-Ping Xu (lead).

## Supplementary Material
