# Supplementary material for: Identifying stage-associated hub genes in bladder cancer via weighted gene co-expression network and robust rank aggregation analyses
Source: Medicine (Baltimore). 2022 Dec 23;101(51):e32318. doi: 10.1097/MD.0000000000032318 (PMC9794320; doi:10.1097/MD.0000000000032318)

Supplementary Figure 1. Study workflow. KEGG: Kyoto Encyclopedia of Genes and Genomes; GSEA: Gene Set Enrichment Analyses GEO: Gene Expression Omnibus; GO: Gene Ontology; TCGA: The Cancer Genome Atlas; TIMER: Tumor Immune Estimation Resource; WGCNA: Weighted Gene Co-expression Network Analysis.

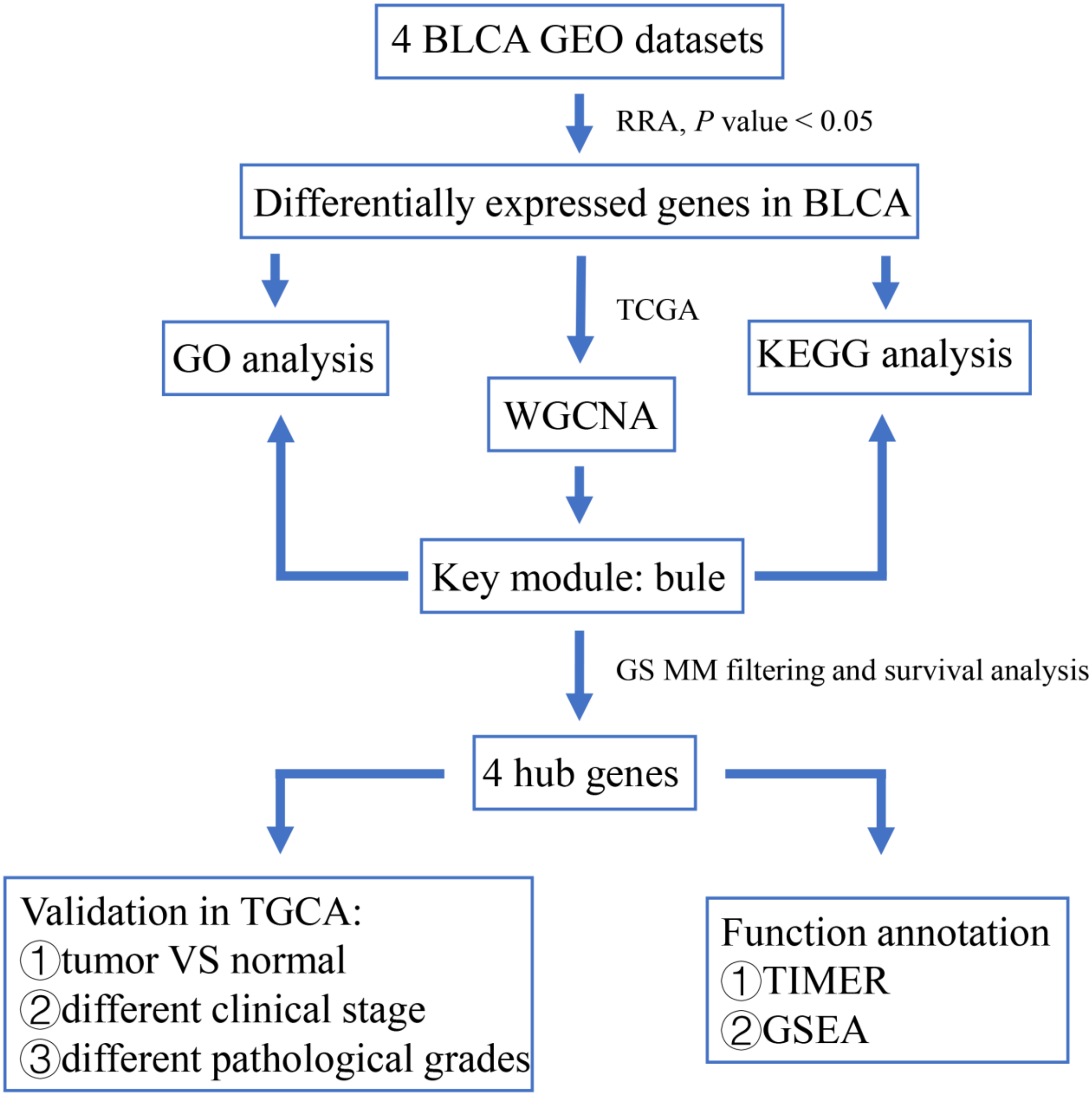

Supplement: Supplementary file 1 [file medi-101-e32318-s001.pdf]
