# Supplementary material for: Identifying stage-associated hub genes in bladder cancer via weighted gene co-expression network and robust rank aggregation analyses
Source: Medicine (Baltimore). 2022 Dec 23;101(51):e32318. doi: 10.1097/MD.0000000000032318 (PMC9794320; doi:10.1097/MD.0000000000032318)

Supplementary Figure 2. Survival analysis of all hub genes in the WGCNA blue module. Kaplan-Meier plots of disease-free survival in two group divided by each hub genes' best-separation value.

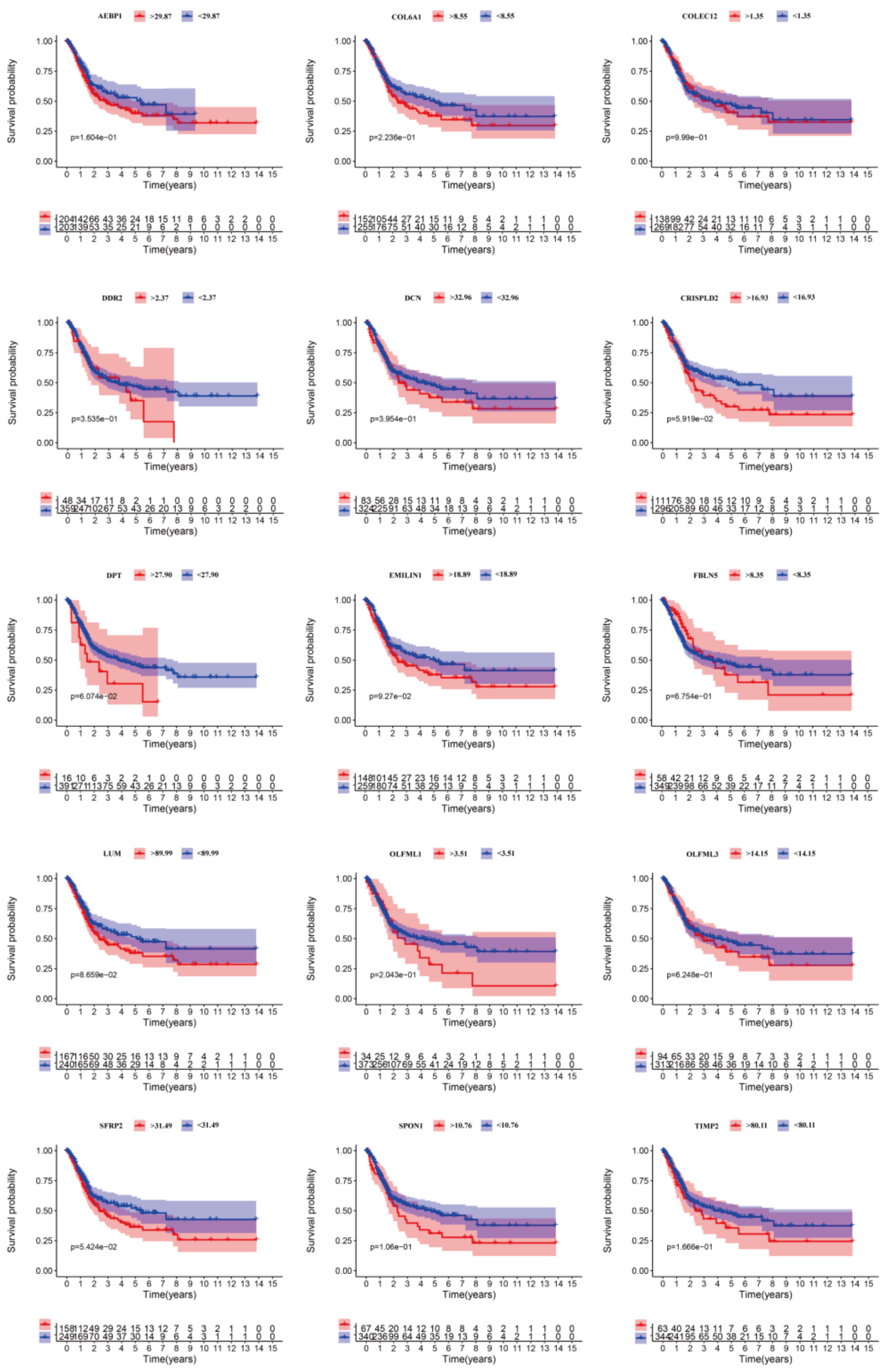

Supplement: Supplementary file 2 [file medi-101-e32318-s002.pdf]
